# Benchmarking the urinary transplant metabolome of kidney transplant recipients using healthy organ donors

**DOI:** 10.3389/frtra.2026.1833811

**Published:** 2026-06-03

**Authors:** Naser B. N. Shehab, Kim C. M. Lammers-Jannink, Margriet H. Riphagen, Siawosh K. Eskandari, Jamil R. Azzi, Martin H. de Borst, Kerstin Bunte, Stephan J. L. Bakker, M. Rebecca Heiner-Fokkema

**Affiliations:** 1 Division of Nephrology, Department of Internal Medicine, University Medical Center Groningen, University of Groningen, Groningen, Netherlands; 2 Intelligent Systems Group, Faculty of Science and Engineering, University of Groningen, Bernoulli Institute for Mathematics, Computer Science and Artificial Intelligence, Groningen, Netherlands; 3 Transplantation Research Center, Division of Nephrology, Department of Internal Medicine, Brigham and Women’s Hospital, Harvard Medical School, Boston, MA, United States; 4 Department of Laboratory Medicine, University Medical Center Groningen, University of Groningen, Groningen, Netherlands

**Keywords:** clinical research, healthy donors, kidney transplantation, transplant recipients, urinary metabolomics

## Abstract

**Background:**

Kidney transplantation is the only curative treatment for end-stage kidney disease, providing markedly improved outcomes over dialysis. In search of optimized long-term outcomes, recent studies have demonstrated alterations in the metabolic state of kidney transplant recipients (KTRs) may contribute to chronic graft outcomes. Here, we sought to benchmark metabolic perturbations associated with the post-transplant state using a set of extensively characterized kidney transplant donors as healthy control.

**Methods:**

In this cross-sectional, single-center study, we used untargeted GC-MS to profile the 24 h urine from 121 stable KTRs (0.2–38.9 yrs post-transplant) and 94 extensively screened healthy potential kidney donors (HC) from the TransplantLines Biobank and Cohort Study. We assessed group differences using linear models adjusted for baseline covariates including eGFR, and the multivariable OPLS-DA model for global separation. ROC analysis was performed on the top discriminants. GlobalTest Pathway enrichment was used to map differentially abundant metabolites in metabolic pathways from the KEGG database.

**Results:**

In KTRs, 28 metabolites were significantly altered compared to HC; 13 of which were elevated and 15 decreased in KTRs. Among them, methylmalonic acid (AUROC 0.886), citric acid (AUROC 0.870), and glycolic acid (AUROC 0.804) were all reduced in KTRs and showed the strongest in-sample separation between the groups. OPLS-DA achieved good group separation (R^2^Y = 0.733, Q^2^ = 0.621) with no signs of overfitting. Twenty pathways were significantly perturbed, led by the TCA cycle, glyoxylate and dicarboxylate metabolism and branched-chain amino acid degradation. Sensitivity analyses examining alternative normalizations, excluding patients with diabetes or proteinuria, adjusting for statin use, and stratifying by eGFR and transplant vintage, were highly concordant with the main analysis (Spearman *ρ* > 0.87 for all pairwise comparisons).

**Conclusion:**

Urinary metabolic profiles in KTRs are consistent with alterations in TCA cycle, glyoxylate and dicarboxylate metabolism, and branched chain amino acid degradation after transplantation. Although causality cannot be established from this cross-sectional design, these findings may reflect altered energy metabolism in the post-transplant state.

## Introduction

While kidney transplantation is the only definitive treatment for patients with end-stage kidney disease, it does not restore normal physiology (1, 2). Instead, it sets a new and persistent baseline that is different from a healthy, untransplanted individual. As a consequence, kidney transplant recipients (KTRs) experience an elevated burden of metabolic and cardiovascular complications compared to the general population (3, 4). Since such systemic changes can threaten long-term allograft health, and late graft loss remains a major challenge in transplantation (5–7), it is critical to understand the drivers of chronic graft pathology. This requires looking beyond routine clinical measures of kidney function, and assessing more granular, underlying cellular and systemic biology.

Untargeted profiling of small-molecule metabolites offers a method for a wide and relatively unbiased exploration of the post-transplant metabolic landscape (8). As the downstream product of genetic, transcriptional, environmental, and pharmacological exposures, the metabolome also provides an integrated and functional snapshot of an individual's physiological state (9, 10). Urine is an especially relevant biofluid in this context; it can be collected non-invasively, reflects both glomerular filtration and tubular metabolic activity, and contains metabolic signatures shaped by systemic biochemical processes (8, 11, 12). This positions it well for detecting chronic, low-grade metabolic shifts associated with the post-transplant state.

Existing applications of urinary metabolomics in transplantation often prioritize differentiating between graft injury states (13–15). While clinically valuable, interpretation of these injury-specific perturbations is fraught and would be strengthened by a more defined baseline of the post-transplant state. Given the generally modest effect sizes and limited sample sizes in high-throughput experiments, even small phenotypic imprecisions can dilute the biological contrast a study is designed to detect (16, 17). Therefore, the quality of the healthy reference group is highly relevant. Prospective living kidney donors from the TransplantLines Biobank are a particularly well-suited reference group. These individuals are extensively screened in a lengthy evaluation process before donation, ensuring a well-characterized and clinically healthy reference group. While living kidney donors differ from KTRs in renal function and comorbidity burden, and cannot substitute for matched non-transplant chronic kidney disease (CKD) controls, factors such as reduced kidney function, chronic medication, and cardiometabolic comorbidity can appropriately be accounted for via covariate adjustment and study design.

Here, we leveraged the TransplantLines Biobank to comprehensively profile the urinary metabolome in pre-donation urine from 94 such healthy potential kidney donors alongside that of 121 KTRs using untargeted gas chromatography-mass spectrometry (GC-MS) analyses. We hypothesized that stable KTRs harbor a distinct urinary metabolic signature associated with the post-transplant state and aimed to characterize these metabolic perturbations and establish a reference baseline for future studies investigating specific graft outcomes.

## Materials and methods

### Study design and patient cohort

This cross-sectional study forms part of the several decades long, ongoing, single-center, prospective TransplantLines Biobank and Cohort Study (clinicaltrials.gov NCT03272841). The study includes (potential) transplant recipients and transplant donors of hearts, lungs, kidneys, livers, and small intestines. From June 2015, all solid organ transplantation patients and transplant donors of the University Medical Center Groningen (UMCG, The Netherlands) were invited to participate. Written informed consent was acquired during enrollment. The overall objective of TransplantLines is to identify and investigate factors related to development of graft failure and to inform patient-centered outcomes in solid organ transplantation. The study is in line with the WMA Declaration of Helsinki and Declaration of Istanbul and received approval from the Medical Ethical Committee of the UMCG (METc 2014/077). Data collection occurred before, during, and at defined intervals after transplantation (that is, at 3, 6, 12, 24, 60 months, and every subsequent five years) for the transplant recipients. For living donors, data were acquired before donation.

The cohort in this study consisted of 121 adult KTRs and 94 living kidney donors, in whom urinary metabolites were measured. All participants underwent clinical and laboratory evaluations pre-donation within the TransplantLines study. Living kidney donors were designated as healthy controls (HC). Included participants visited the UMCG outpatient clinic between September 2015 and November 2017. Trained nursing staff collected data during morning screening visits at the UMCG, including blood, feces, nails, and hair samples. Pooled 24-hour urine samples were collected according to a strict standardized protocol, as previously described (18). Participants were instructed to discard the first morning void on the day before the clinic visit, collect all subsequent urine over the next 24 h and include the following morning's first void. Potential inadequacies in 24 h urine collections were defined as a difference of more than 2.5 standard deviations between estimated and measured urine volumes. The estimated urine volume was derived from the formula creatinine clearance = [(urinary creatinine) x 24-hour urine volume]/[serum creatinine], where creatinine clearance was estimated using the Cockcroft-Gault formula (19). Participants with potentially inadequate collections were excluded as part of a sensitivity analysis. KTRs were eligible if they were ≥18 years of age. Participants were excluded in the event of known or overt systemic illness, history of drug or alcohol addiction, inability to communicate in Dutch or inability to comprehend study questionnaires. KTRs were considered clinically stable at the time of sampling if confirmed by an outpatient visit without active rejection, acute infection or recurrent disease, as described previously (18). The 94 healthy controls were potential kidney donors who had completed the standard UMCG donor screening protocol.

### Gas chromatography-mass spectrometry (GC-MS)

For urinary metabolite evaluation, a 1 mL urine aliquot was combined with an internal control solution of 4-phenylbutyric acid (0.5 mg/mL in 0.1M HCl, 100 μL) and ethoxy-amine HCl solution (200 mg/mL in H_2_O, 100 μL). This mixture was then derivatized in glass containers at 60°C for 30 min. Following the addition of a spatula tip of NaCl and two drops of 37% HCl, the mixture was extracted using a 1:1v/v solution of ethyl-acetate/diethyl-ether. The organic fraction containing the derivatized acids was isolated and evaporated under a vacuum. Next, a 200 μL aliquot of derivatizing reagent consisting of BSTFA, pyridine, and trichloro-methyl-silane (5/1/0.06v/v/v) was introduced for a second derivatization process at 60°C for 30 min.

Prepared samples (1 µL) were injected into the GC-MS. Organic acids were analyzed using a Thermo Scientific Trace 1,310 gas chromatograph coupled to an ISQ LT single quadrupole mass spectrometer (Interscience, Breda, The Netherlands), operated in EI positive ionization mode at 70 eV and registering a total ion scan (m/z range 50–650). Separation was achieved using a (14% cyanopropylphenyl) methylpolysiloxan column (30 m × 0.250 mm × 0.25 µm film thickness; Restek Chromatography, Bellefonte, PA, USA), with helium as the carrier gas (0.8 mL/min).

Data compilation and quantitative evaluations were performed using Chromeleon Version 7.2 SR4 software. Untargeted GC-MS metabolic profiles were pre-processed with PyChromics, a python-based pre-processing tool developed by the UMCG and Rijksuniversiteit Groningen specifically designed for organic acid assessments, to prepare the data for downstream statistical analysis. Concurrently, an enzymatic analysis was conducted on the same samples to quantify creatinine levels using a Roche Clinical Chemistry Analyzer (Cobas, Roche Diagnostics, Mannheim, Germany). Quantitative results were computed by dividing the target compound area by the internal control area. Finally, data were normalized to the corresponding urinary creatinine concentrations to account for variations in urine water content. Metabolite values that fell below the limit of detection (signal area <0.00043) were substituted with random noise values within the sub-detection range. Metabolite identification was based on GC-MS spectral library matching, consistent with putative annotation. No technical replicates were performed. Quality control samples at two concentration levels were analyzed across the measurement period to monitor analytical reproducibility (Supplementary Table S1). Batch effects were assessed by principal component analysis; visual inspection of the scores showed no meaningful batch-related clustering (Supplementary Figure S1).

### Statistical and bioinformatics analysis

Baseline characteristics of the study cohort were summarized and compared between KTRs and HC. Depending on data distribution, continuous variables were presented as mean ± standard deviation (SD) or median [Q_1_–Q_3_] and compared using a Student's *t*-test or Mann–Whitney *U*-test, respectively. Categorical variables were expressed as proportions and compared using the *χ*^2^-test or Fisher's exact test as appropriate.

Univariable analyses were performed to identify differentially abundant urinary metabolites between groups. Specifically, we calculated fold changes and false discovery rate (FDR)-adjusted *p*-values using the Benjamini-Hochberg procedure and visualized them using volcano plots. We evaluated the in-sample discriminatory ability of individual metabolites by calculating the area under the receiver operating characteristic (AUROC) curve; these values reflecting within-sample group separation only, which should not be interpreted as validated diagnostic performance. Then, we constructed linear models to account for potential confounders and to assess metabolite significance before and after adjusting for relevant baseline clinical covariates. Covariates were selected *a priori* based on clinical relevance. Age, sex and BMI were included as standard demographic potential confounders. eGFR was included because it may affect urinary metabolite excretion and is expected to differ between KTRs and healthy subjects. Other baseline characteristics that differed significantly between groups were additionally included as covariates. Diabetes was included given its known influence on metabolic pathways. Transplant vintage, immunosuppressive regimen and donor type were not included as covariates, as these variables only apply to KTRs and cannot be modeled in a between-group comparison. Additional sensitivity analyses adjusting for statin use, analyzing metabolite excretions and concentrations not normalized for creatinine, excluding patients with diabetes or proteinuria, and stratified analyses by eGFR and transplant vintage within KTRs were performed to assess the robustness of our findings. FDR correction using the Benjamini-Hochberg procedure was applied to both adjusted and unadjusted models. Metabolite values were log_2_-transformed and scaled prior to analysis; distributions were visually assessed and found to approximate normality.

Next, we applied a supervised multivariable pattern recognition algorithm to maximize separation between KTRs and HC groups. Specifically, the orthogonal projections to latent structures discriminant analysis (OPLS-DA) model was used. OPLS-DA was implemented in R using the ropls package with one predictive component, the number of orthogonal components was determined via 7-fold cross-validation. Model-level significance was assessed by permutation testing with 1,000 permutations. To evaluate feature importance and ranking stability, VIP scores were independently extracted from models trained within a separate 5-fold cross-validation scheme and averaged across folds. A hierarchically clustered heatmap was used to visualize the top discriminative features across samples. Finally, to provide biological context to the observed metabolic shifts, pathway enrichment analysis was applied. We mapped metabolites to known biochemical networks in the KEGG database using the GlobalTest algorithm implemented in MetaboAnalystR. Pathway impact and significance were assessed to identify heavily perturbed networks and to contextualize important discriminant features with established pathways. All statistical analyses and data visualizations were performed using R software (version 4.4.0).

## Results

### Baseline characteristics of the study population

Our study included a total of 215 individuals, comprising 121 KTRs and 94 HC. The baseline clinical and demographic characteristics are summarized in Table 1. The two groups were comparable across age [60.0 [54.3, 67.0] vs. 61.0 [53.0, 67.0] years, *p* = 0.81], sex distribution (59.6% vs. 51.2% male, *p* = 0.28), and BMI (27.1 ± 3.6 vs. 27.3 ± 5.5 kg/m^2^, *p* = 0.72). Consistent with the clinical profile of this population, KTRs had a significantly lower renal function compared to HC, evidenced by a lower eGFR (49.6 ± 16.9 vs. 66.5 ± 17.2 mL/min/1.73m^2^, *p* < 0.001). Furthermore, KTRs demonstrated higher levels of systemic inflammation [C-reactive protein; 1.3 [0.7, 3.0] vs. 2.2 [1.1, 4.6] mg/L, *p* = 0.019] and elevated systolic blood pressure (141.7 ± 19.9 vs. 133.4 ± 16.0 mmHg, *p* = 0.001). The prevalence of comorbid diabetes (30.6% vs. 2.1%, *p* < 0.001) and the use of statins (52.1% vs. 12.1%, *p* < 0.001) were also substantially higher in the KTRs cohort. Within the KTRs group, most patients were maintained on standard immunosuppressive regimens, including prednisolone (97.5%), proliferation inhibitors (81.0%), and calcineurin inhibitors (76.0%).

**Table 1:** Baseline patient characteristics.

Characteristic^a^	Overall (*n* = 215)	HC (*n* = 94)	KTR (*n* = 121)	*P*-value^b^
Male, *n* (%)	118 (54.9)	56 (59.6)	62 (51.2)	0.28
Age, yrs	61.0 [54.0, 67.0]	60.0 [54.3, 67.0]	61.0 [53.0, 67.0]	0.81
BMI, kg/m^2^	27.2 (4.8)	27.1 (3.6)	27.3 (5.5)	0.72
eGFR, mL/min/1.73 m²	57.0 (19.0)	66.5 (17.2)	49.6 (16.9)	**<0**.**001**
CRP, mg/L	1.8 [0.8, 3.9]	1.3 [0.7, 3.0]	2.2 [1.1, 4.6]	**0**.**019**
Systolic BP mmHg	138.1 (18.7)	133.4 (16.0)	141.7 (19.9)	**0**.**001**
Diastolic BP, mmHg	78.9 (10.8)	77.7 (11.1)	79.9 (10.5)	0.14
Post-transplant time, yrs	5.89 [1.34, 12.14]	–	5.89 [1.34, 12.14]	–
Diabetes, *n* (%)	39 (18.1)	2 (2.1)	37 (30.6)	**<0**.**001**
Proteinuria, >0.5 g/24h	20 (11.6)	–	20 (20.4)	–
Calcineurin inhibitors, *n* (%)	92 (43.4)	–	92 (76.0)	–
Proliferation inhibitors, *n* (%)	98 (46.2)	–	98 (81.0)	–
Prednisolone, *n* (%)	118 (55.7)	–	118 (97.5)	–

a Data are presented as mean (SD) for normally distributed continuous variables, median [IQR] for non-normally distributed continuous variables, and *n* (%) for categorical variables.

b *p*-values compare HC vs. KTR groups using Student's *t*-test for normally distributed variables, Mann–Whitney *U*-test for non-normally distributed variables, and Chi-square test (or Fisher's exact test for small cell counts) for categorical variables. A two-sided *p*-value of <0.05 was considered statistically significant.

Bold values indicate statistical significance *p* < 0.05.

### Urinary metabolome is significantly altered after transplantation

**Figure 1:**
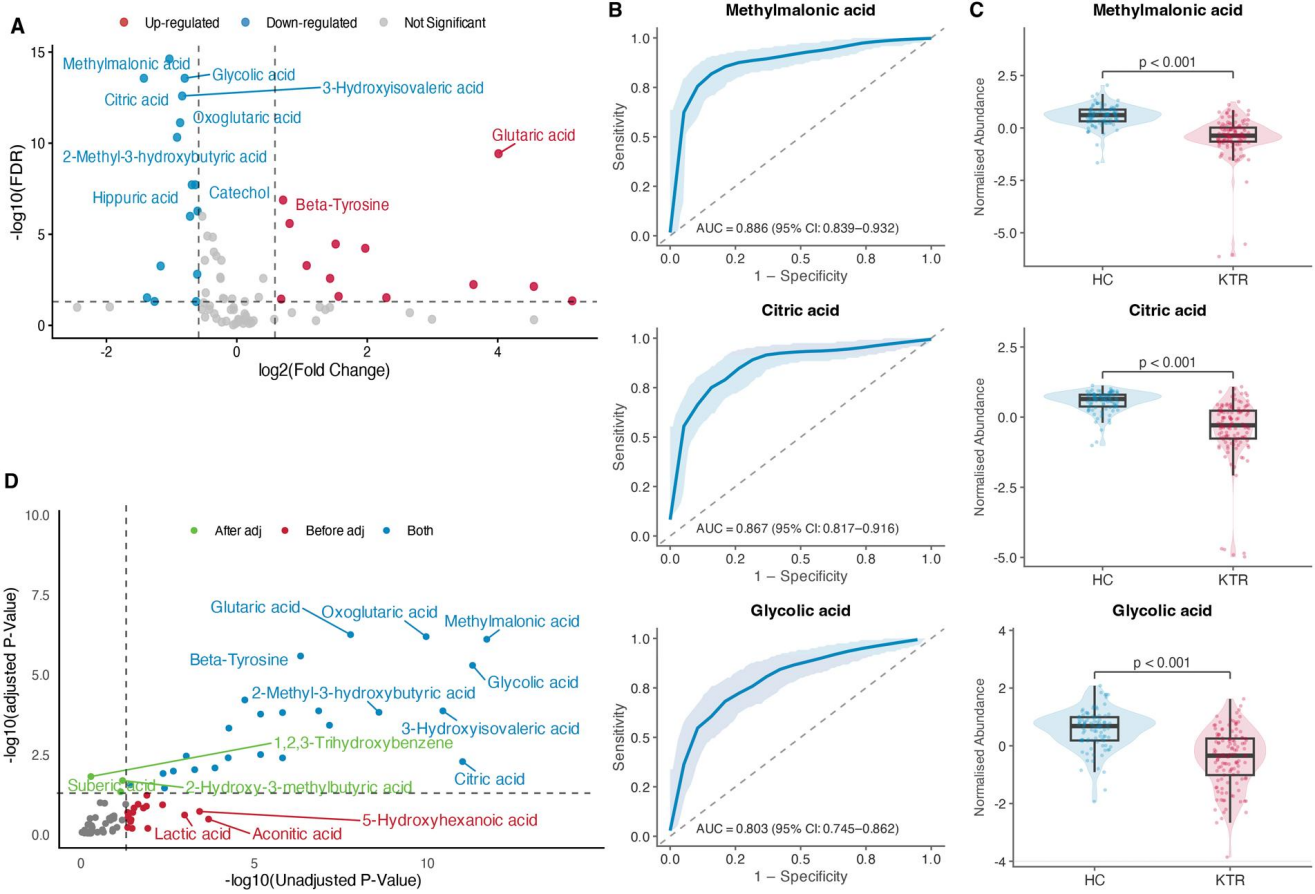
Univariable analyses identify differentially abundant metabolites in KTRs. Volcano plot of all detected metabolites, with significantly altered metabolites (|FC|≥1.5,FDR < 0.05) **(A)**, ROC curves for the top discriminant metabolites **(B)**, boxplots of creatinine-normalized urinary excretion for the top discriminant metabolites across groups **(C)**, and scatter plot of metabolite significance before [on x-axis] vs. after [on y-axis] adjustment for age, sex, BMI, eGFR, systolic blood pressure, CRP, and diabetes mellitus **(D)**.

We conducted an initial univariable differential analysis between KTRs and HC to identify the primary metabolic perturbations that distinguish a transplanted individual from a healthy population. This analysis identified 28 metabolites that were significantly altered under an FDR-adjusted *p* < 0.05 and an absolute fold change (FC) ≥1.5 (Figure 1A). Of those, 13 metabolites were increased and 15 were decreased in KTRs. AUROC curves and boxplots subsequently demonstrated a more detailed view for the most discriminant metabolites (Figures 1B,C). Top hits included methylmalonic acid (mean in-sample AUROC = 0.886), citric acid (mean in-sample AUROC = 0.870), and glycolic acid (mean in-sample AUROC = 0.804), which were all downregulated in KTRs. These values notably require external validation before any inference can be made on clinical utility. In an additional differential analysis using linear regression models, with adjustment for sources of potential confounding, including age, sex, BMI, eGFR, systolic blood pressure, CRP, and diabetes status, we assessed the independence of the metabolites. Although minor changes were observed, the overall shift, and specifically the most prominent hits, remained highly significant and unaffected (Figure 1D, Table 2). Three metabolites (1,2,3-trihydroxybenzene, 2-hydroxy-3-methylbutyric acid, and suberic acid) reached significance only after covariate adjustment, likely consistent with negative confounding.

**Table 2:** Linear models.

Metabolite	Status	Unadjusted model			Adjusted model		
		log₂FC	95% CI	FDR	log₂FC	95% CI	FDR
Glutaric acid	Both	0.814	(0.553, 1.074)	0.000	0.907	(0.611, 1.202)	0.000
Oxoglutaric acid	Both	−0.923	(−1.182, −0.663)	0.000	−0.888	(−1.189, −0.588)	0.000
Methylmalonic acid	Both	−1.026	(−1.284, −0.768)	0.000	−0.865	(−1.159, −0.572)	0.000
Beta-Tyrosine	Both	0.730	(0.468, 0.992)	0.000	0.834	(0.529, 1.138)	0.000
Glycolic acid	Both	−0.997	(−1.255, −0.739)	0.000	−0.788	(−1.073, −0.504)	0.000
2-Hydroxybutyric acid	Both	−0.624	(−0.887, −0.361)	0.000	−0.715	(−1.011, −0.419)	0.000
3-Hydroxyisovaleric acid	Both	−0.948	(−1.207, −0.689)	0.000	−0.672	(−0.958, −0.386)	0.000
Hippuric acid	Both	−0.761	(−1.022, −0.499)	0.000	−0.686	(−0.987, −0.384)	0.000
2-Methyl−3-hydroxybutyric acid	Both	−0.857	(−1.117, −0.597)	0.000	−0.674	(−0.974, −0.375)	0.000
p-Hydroxyhippuric acid	Both	0.696	(0.434, 0.958)	0.000	0.676	(0.369, 0.983)	0.000
Hydroxypropionic acid	Both	−0.654	(−0.917, −0.392)	0.000	−0.665	(−0.968, −0.363)	0.000
Catechol	Both	−0.780	(−1.041, −0.518)	0.000	−0.632	(−0.934, −0.331)	0.000
3-Hydroxysebacic acid	Both	0.592	(0.329, 0.855)	0.000	0.624	(0.319, 0.930)	0.000
(S)-3-Hydroxyisobutyric acid	Both	−0.557	(−0.820, −0.293)	0.000	−0.557	(−0.851, −0.262)	0.002
3-Hydroxyphenylacetic acid	Both	−0.656	(−0.919, −0.394)	0.000	−0.540	(−0.843, −0.238)	0.003
Pyruvic acid	Both	−0.491	(−0.755, −0.227)	0.001	−0.539	(−0.852, −0.227)	0.003
2-Ethylhydracrylic acid	Both	−0.589	(−0.852, −0.326)	0.000	−0.526	(−0.829, −0.222)	0.004
Ethylmalonic acid	Both	−0.697	(−0.959, −0.435)	0.000	−0.521	(−0.821, −0.220)	0.004
Citric acid	Both	−0.978	(−1.236, −0.719)	0.000	−0.498	(−0.784, −0.212)	0.005
4-Hydroxybenzoic acid	Both	0.561	(0.297, 0.824)	0.000	0.485	(0.181, 0.789)	0.008
Malic acid	Both	−0.511	(−0.775, −0.248)	0.001	−0.480	(−0.790, −0.171)	0.009
2-Furoylglycine	Both	−0.458	(−0.722, −0.193)	0.002	−0.474	(−0.785, −0.163)	0.010
Succinic acid	Both	0.427	(0.163, 0.692)	0.004	0.461	(0.154, 0.768)	0.012
Urea	Both	−0.317	(−0.582, −0.052)	0.038	−0.413	(−0.718, −0.107)	0.027
4-Hydroxycyclohexylcarboxylic acid	Both	−0.433	(−0.697, −0.168)	0.004	−0.401	(−0.716, −0.087)	0.035
4-Deoxyerythronic acid	Before adj	−0.379	(−0.644, −0.115)	0.013	−0.366	(−0.676, −0.055)	0.058
3-(3-Hydroxyphenyl)propanoic acid	Before adj	0.349	(0.084, 0.614)	0.023	0.318	(0.005, 0.630)	0.111
4-Deoxythreonic acid	Before adj	−0.425	(−0.689, −0.160)	0.004	−0.308	(−0.607, −0.009)	0.116
3-Hydroxybenzoic acid	Before adj	0.376	(0.112, 0.641)	0.013	0.304	(−0.009, 0.618)	0.129
4-hydroxyvaleric acid	Before adj	−0.366	(−0.631, −0.102)	0.016	−0.293	(−0.605, 0.020)	0.146
Salicyluric acid	Before adj	0.335	(0.070, 0.600)	0.030	0.291	(−0.022, 0.605)	0.146
5-Hydroxyhexanoic acid	Before adj	−0.524	(−0.787, −0.260)	0.000	−0.269	(−0.576, 0.037)	0.187
Ketoleucine	Before adj	−0.330	(−0.595, −0.065)	0.032	−0.262	(−0.575, 0.050)	0.201
2-Methylbutyrylglycine	Before adj	0.303	(0.038, 0.568)	0.045	0.263	(−0.053, 0.579)	0.201
Phenoxyacetic acid	Before adj	−0.309	(−0.574, −0.044)	0.043	−0.243	(−0.543, 0.058)	0.240
Lactic acid	Before adj	0.486	(0.222, 0.750)	0.001	0.242	(−0.064, 0.547)	0.242
Isocitric acid	Before adj	−0.303	(−0.568, −0.038)	0.045	−0.216	(−0.531, 0.099)	0.308
(S)-3,4-Dihydroxybutyric acid	Before adj	−0.446	(−0.710, −0.182)	0.003	−0.203	(−0.502, 0.096)	0.316
Aconitic acid	Before adj	−0.544	(−0.808, −0.281)	0.000	−0.198	(−0.497, 0.100)	0.325
Acetoacetic acid	Before adj	−0.321	(−0.586, −0.057)	0.036	−0.197	(−0.512, 0.117)	0.337
p-Cresol	Before adj	−0.319	(−0.584, −0.054)	0.037	−0.178	(−0.486, 0.130)	0.385
Vanillylmandelic acid	Before adj	−0.303	(−0.568, −0.038)	0.045	−0.119	(−0.427, 0.188)	0.592
Hydroxyphenyllactic acid	Before adj	0.383	(0.119, 0.648)	0.012	0.104	(−0.194, 0.403)	0.633
3-Methylglutaconic acid	Before adj	0.326	(0.061, 0.591)	0.034	0.107	(−0.208, 0.422)	0.633
1,2,3-Trihydroxybenzene	After adj	0.101	(−0.164, 0.367)	0.521	0.451	(0.138, 0.765)	0.015
2-Hydroxy-3-methylbutyric acid	After adj	−0.282	(−0.547, −0.017)	0.063	−0.435	(−0.749, −0.121)	0.020
Suberic acid	After adj	−0.273	(−0.538, −0.008)	0.072	−0.385	(−0.702, −0.069)	0.045

Bold values indicate statistical significance FDR < 0.05.

### Supervised OPLS-DA globally separates kidney transplant recipients and healthy controls

**Figure 2:**
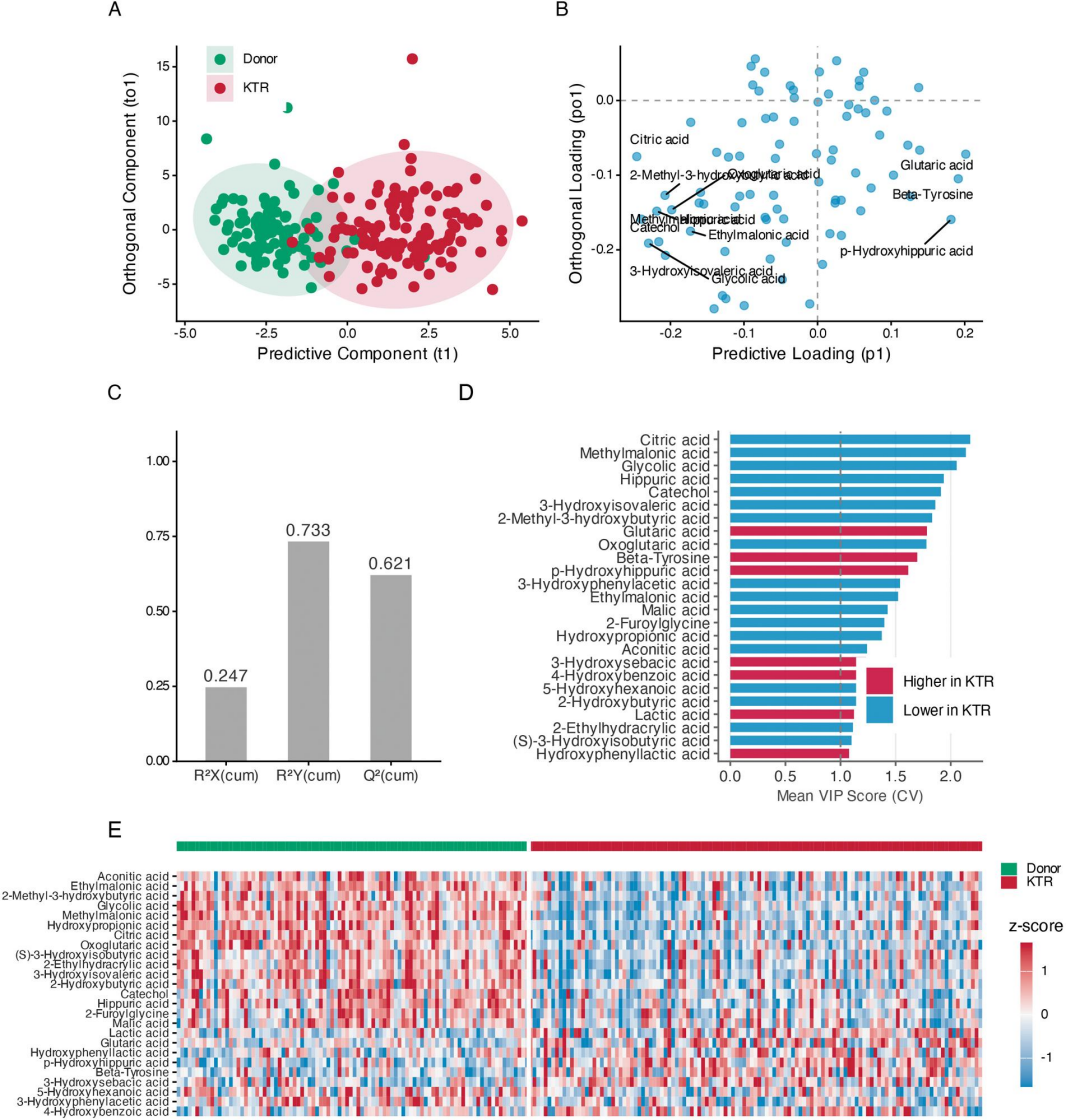
OPLS-DA globally separates kidney transplant recipients from healthy controls. Scores plot showing group separation along predictive component **(A)**, loadings plot illustrating metabolite contribution to group separation **(B)**, model performance metrics **(C)**, cross-validated VIP scores for the top 25 metabolites **(D)**, and hierarchically clustered heatmap of the top 25 VIP-ranked metabolites across all individuals **(E)**.

To evaluate systemic metabolic shifts through an integrated analysis of the metabolome, while accounting for inherent multicollinearity of metabolic networks, we trained and evaluated a supervised OPLS-DA model with one predictive component and automatic selection of orthogonal components. The final model retained two orthogonal components. The scores plot derived from this model showed a clear metabolic separation of KTRs and HC (Figure 2A). The corresponding loadings plot shows the contribution of individual metabolites to this separation (Figure 2B). The final model had an R^2^Y of 0.733 and a Q^2^ of 0.621 (permutation *p* = 0.001, Figure 2C). We then examined cross-validated VIP scores for stability across folds to identify the metabolites contributing most strongly to group separation (Figure 2D). Consistent with the independent univariable and multivariable linear models, methylmalonic acid, citric acid, and glycolic acid were the most prominent, though not the sole, markers of this metabolic shift. Finally, a hierarchically clustered heatmap of the top 25 VIP features was generated to visualize their relative abundance across individuals and groups (Figure 2E).

### Transplantation significantly disturbs the urinary metabolome

**Figure 3:**
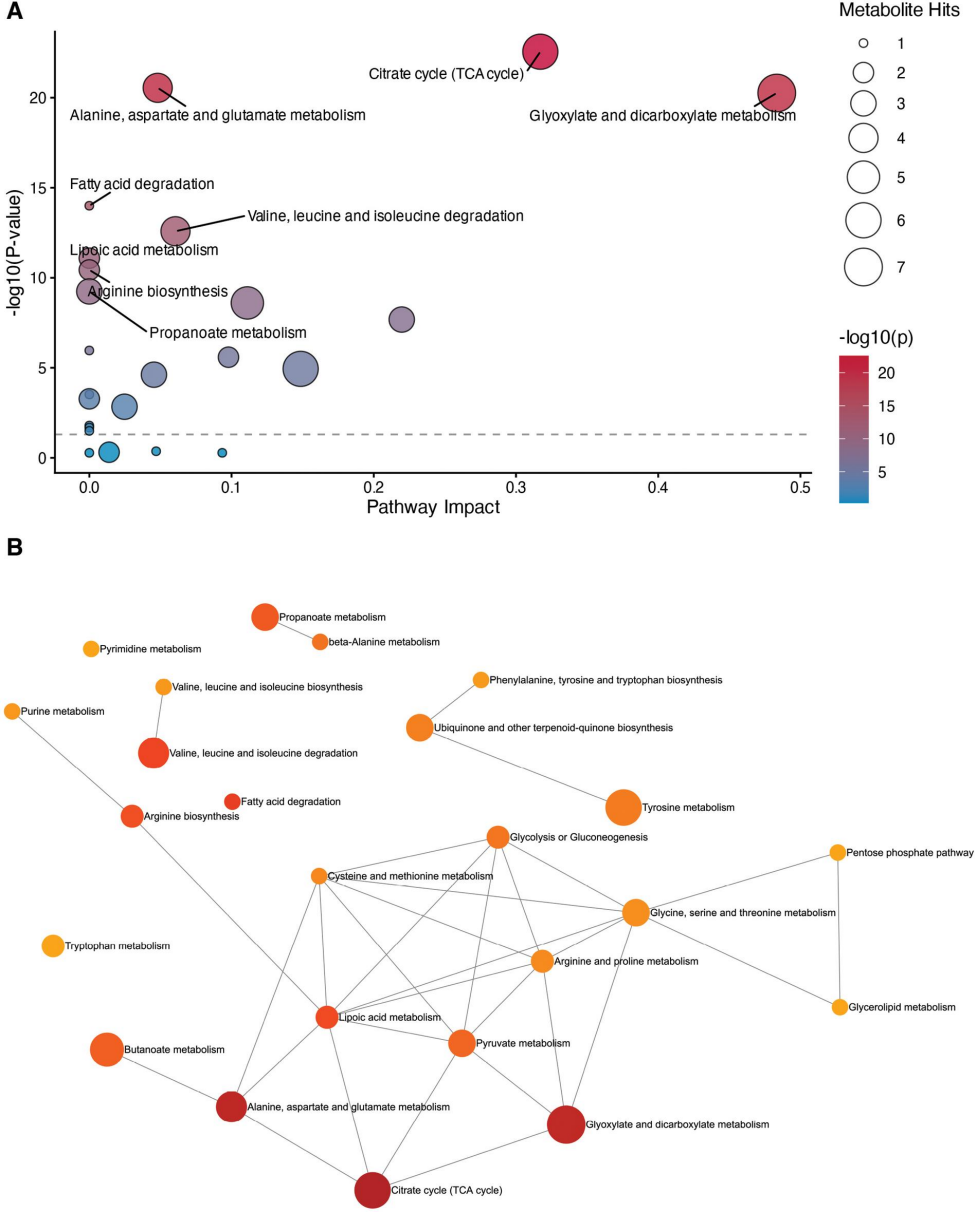
Pathway enrichment analysis reveals coordinated perturbations of metabolic networks. Scatter plot of enriched KEGG pathways by topological impact and statistical significance (FDR < 0.05); point size reflects the number of metabolite hits **(A)**, and network diagram of enriched pathways connected by edges when sharing ≥5 pathway members **(B)**.

To contextualize the identified metabolic shifts within a broader systems biology framework, we performed pathway enrichment analysis. We used the GlobalTest algorithm to evaluate coordinated differential abundance across metabolite sets mapped to curated KEGG pathways (Figure 3A). The *x*-axis represents pathway impact, a topological metric reflecting the centrality of detected hits within the pathway network architecture, while the *y*-axis represents statistical significance after FDR correction.

In total, 20 pathways reached significance at FDR < 0.05 (Table 3, Figure 3A). Among these, the strongest statistical enrichment was observed for the *citrate cycle* (TCA cycle; 6 hits out of 20 mapped metabolites; FDR = 6.69 × 10^−22^), *alanine, aspartate and glutamate metabolism* (4/28; FDR=3.39 × 10^−20^), and *glyoxylate and dicarboxylate metabolism* (7/32; FDR =4.38 × 10^−20^). Other notably enriched pathways included *fatty acid degradation* (1/39; FDR = 5.98 × 10^−14^), *valine, leucine and isoleucine degradation* (4/40; FDR = 1.25 × 10^−12^), *propanoate* metabolism (3/22; FDR = 1.72 × 10^−9^), *butanoate* metabolism (5/15; FDR = 6.64 × 10^−9^), and *pyruvate metabolism* (3/23; FDR = 5.01 × 10^−8^). It must be noted that pathways supported by a few annotated metabolite hits or negligible topological impact should be interpreted with caution; both metrics are reported for all pathways in Table 3. Next, we visualized the network of pathway interconnectivity, with edges drawn when ≥5 of pathway members were shared (Figure 3B).

**Table 3:** Pathway analysis results.

Pathway	Total	Hits	P	FDR	Impact
Citrate cycle (TCA cycle)	**20** **.** **000**	**6**.**000**	**0**.**000**	**0**.**000**	**0**.**317**
Alanine, aspartate and glutamate metabolism	**28**.**000**	**4**.**000**	**0**.**000**	**0**.**000**	**0**.**048**
Glyoxylate and dicarboxylate metabolism	**32**.**000**	**7**.**000**	**0**.**000**	**0**.**000**	**0**.**483**
Fatty acid degradation	**39**.**000**	**1**.**000**	**0**.**000**	**0**.**000**	**0**.**000**
Valine, leucine and isoleucine degradation	**40**.**000**	**4**.**000**	**0**.**000**	**0**.**000**	**0**.**061**
Lipoic acid metabolism	**28**.**000**	**2**.**000**	**0**.**000**	**0**.**000**	**0**.**000**
Arginine biosynthesis	**14**.**000**	**2**.**000**	**0**.**000**	**0**.**000**	**0**.**000**
Propanoate metabolism	**22**.**000**	**3**.**000**	**0**.**000**	**0**.**000**	**0**.**000**
Butanoate metabolism	**15**.**000**	**5**.**000**	**0**.**000**	**0**.**000**	**0**.**111**
Pyruvate metabolism	**23**.**000**	**3**.**000**	**0**.**000**	**0**.**000**	**0**.**220**
beta-Alanine metabolism	**21**.**000**	**1**.**000**	**0**.**000**	**0**.**000**	**0**.**000**
Glycolysis or Gluconeogenesis	**26**.**000**	**2**.**000**	**0**.**000**	**0**.**000**	**0**.**098**
Tyrosine metabolism	**42**.**000**	**6**.**000**	**0**.**000**	**0**.**000**	**0**.**149**
Ubiquinone and other terpenoid-quinone biosynthesis	**19**.**000**	**3**.**000**	**0**.**000**	**0**.**000**	**0**.**045**
Cysteine and methionine metabolism	**33**.**000**	**1**.**000**	**0**.**000**	**0**.**000**	**0**.**000**
Arginine and proline metabolism	**36**.**000**	**2**.**000**	**0**.**001**	**0**.**001**	**0**.**000**
Glycine, serine and threonine metabolism	**33**.**000**	**3**.**000**	**0**.**001**	**0**.**002**	**0**.**025**
Valine, leucine and isoleucine biosynthesis	**8**.**000**	**1**.**000**	**0**.**016**	**0**.**021**	**0**.**000**
Purine metabolism	**70**.**000**	**1**.**000**	**0**.**021**	**0**.**026**	**0**.**000**
Phenylalanine, tyrosine and tryptophan biosynthesis	**4**.**000**	**1**.**000**	**0**.**032**	**0**.**038**	**0**.**000**
Pyrimidine metabolism	39.000	1.000	0.429	0.490	0.047
Tryptophan metabolism	41.000	2.000	0.487	0.525	0.014
Glycerolipid metabolism	16.000	1.000	0.525	0.525	0.093
Pentose phosphate pathway	23.000	1.000	0.525	0.525	0.000

When considering topological impact alongside statistical significance, *glyoxylate and dicarboxylate metabolism* were the most prominent across all enriched pathways with an impact score of 0.483 (Supplementary Figure S2). The primary contributing metabolite was glycolic acid, the third ranked individual discriminant in both univariable and multivariable analyses. The *TCA cycle* enrichment was driven predominantly by citric acid and oxoglutaric acid (Supplementary Figure S3), both identified as significant markers in all previous analyses. Methylmalonic acid, the top-ranked discriminant overall, contributed significantly to the enrichment of the BCAA degradation pathway (Supplementary Figure S4). The remaining enriched pathways returned relatively negligible to modest topological impact scores.

### Sensitivity analyses

**Figure 4:**
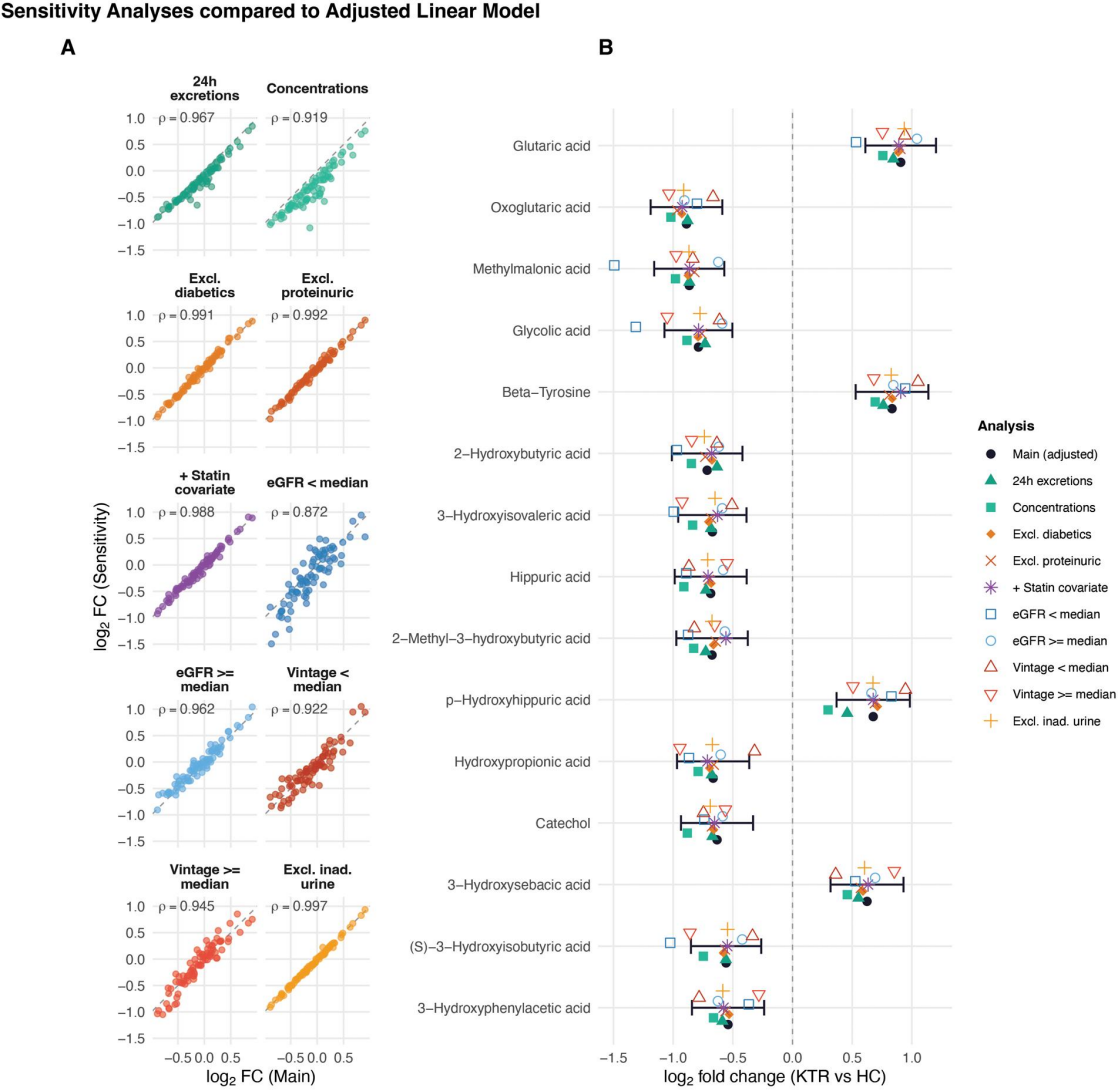
*Sensitivity analyses.* Sensitivity analyses confirm the stability of differential metabolite signature. Pairwise scatter plots of log2 fold changes from the main adjusted model (*x*-axis) against each of the ten sensitivity analyses (*y*-axis) **(A)**, and forest plot of log2 fold changes with 95% confidence intervals for the top discriminant metabolites across the main and sensitivity models **(B)**.

To assess the stability of our findings, we performed multiple sensitivity analyses. We repeated the adjusted differential analysis after excluding KTRs with diabetes (*n* = 37), after excluding KTRs with proteinuria (*n* = 20), after excluding potentially inadequate urine collection samples (*n* = 3), and after additional adjustment for statin use. We additionally analyzed absolute metabolite excretion rates and urinary concentrations instead of creatinine-normalized values, and stratified KTRs by eGFR and transplant vintage (below vs. above median). Across all sensitivity analyses, effect size estimates were highly concordant with the main analysis (Spearman *ρ* > 0.87 for all pairwise comparisons of log_2_-fold changes), and the top discriminant metabolites remained consistent (Figure 4).

## Discussion

Kidney transplantation alters the metabolic profile of recipients, and a growing body of evidence links the post-transplant state to elevated cardiometabolic risk and chronic graft dysfunction (3, 4). Much remains to be learned about the nature and the drivers of the metabolic changes associated with kidney transplantation and their downstream effects (8, 20). In this study, we used an extensively screened cohort of living kidney donors as a healthy reference group to benchmark the urinary metabolome in KTRs. Our findings reveal that the metabolic signature of KTRs and healthy individuals quantifiably differs and the distinctions persist after adjustment for major clinical covariates.

Specifically, we identified 28 metabolites that differed significantly between groups (FDR < 0.05, FC ≥ 1.5), of which 13 were decreased and 15 increased in KTRs. Across the analyses, three metabolites consistently emerged as the strongest discriminants: methylmalonic acid, citric acid, and glycolic acid. All three metabolites showed a reduced concentration in the urine of KTRs. The OPLS-DA model demonstrated a clear metabolic separation between KTRs and healthy controls and had strong performance metrics (R2Y = 0.733, Q2 = 0.621; permutation *p* = 0.001), indicating a stable and reproducible signal and reinforcing the robustness of the metabolic signature. Pathway enrichment analysis showed that differential metabolites clustered within coherent biochemical routes. The most prominent pathways included the TCA cycle, glyoxylate and dicarboxylate metabolism, and BCAA degradation.

Among the enriched pathways, the TCA cycle showed the strongest statistical enrichment. Pyruvic acid and the TCA cycle intermediates citric acid, 2-oxoglutaric acid, and malic acid were reduced in KTRs. Succinic acid, however, was elevated. Persistence of these changes after adjusting for potential clinical confounders argue against a purely filtration-dependent explanation, though the underlying basis cannot be established based on this observational data alone. Our findings share similarities with those in non-diabetic CKD patients, for which Hallan et al. reported comparable reductions in the urinary excretion of these metabolites, and a decreased expression of ten TCA cycle enzymes in kidney tissue (21). Separate studies in KTRs similarly observed lower circulating citric acid and pyruvic acid (22). Though speculative, that these patterns persist after transplantation is consistent with the possibility that mitochondrial energy metabolism is not fully restored by engraftment and could reflect long-lasting injury from the transplantation process or ongoing metabolic stress within the graft (23, 24). It should be acknowledged that the overlap with findings in non-transplant CKD suggests that these TCA cycle perturbations may partly reflect reduced renal mass or chronic kidney injury rather than transplant-specific changes. The notion of overlap between CKD and transplantation warrants the further assessment of these observations in future work to understand the distinctions between the metabolomic profile of renal pathology and the transplant process.

Next, the elevation of succinate stands in contrast to the broader pattern of TCA intermediate reductions and notably diverges from what Hallan et al. found in non-diabetic CKD. In their study, they reported an overall reduction in TCA cycle metabolites, including decreased urinary succinate (21). This suggests that an elevation of succinate in our KTRs cohort may reflect a mechanism more specific to the transplantation context. One possible explanation involves ischemia-reperfusion injury (IRI). In experimental models of IRI, succinate dehydrogenase has been shown to operate in reverse during hypoxia, reducing fumarate to succinate. Upon reperfusion, rapid reoxidation of accumulated succinate by complex II has been reported to drive electron leakage and reactive oxygen species generation at complex I. This burst of ROS has been shown to inflict oxidative damage on proteins, lipids, and DNA (25–28). More broadly, succinate accumulation is thought to reflect an imbalance between oxygen supply and metabolic demand, where its buildup is driven by perturbations in the electron transport chain or other metabolic stress (29). Elevated succinate in KTRs may be consistent with ongoing mitochondrial stress beyond the perioperative period, though this interpretation requires direct experimental validation. Notably, succinate has also been reported to function as a signaling molecule through the renal receptor SUCNR1, where it has been implicated in inflammatory and immune responses (30, 31). Whether elevated succinate in our cohort relates to mitochondrial dysfunction, SUCNR1-mediated inflammatory signaling, or both, cannot be determined from this study.

Perturbations in BCAA catabolism and fatty acid *β*-oxidation align with the broader pattern of metabolic changes observed in this cohort, which together are consistent with altered metabolic flux. MMA, the most discriminant metabolite in our cohort, was significantly reduced in KTRs, its production stemming from the mitochondrial conversion of propionyl-CoA, linking the degradation of BCAAs and odd-chain fatty acids to the TCA cycle via succinyl-CoA. Reduced concentrations of MMA, 3-hydroxyisovaleric acid and 2-methyl-3-hydroxybutyric acid may also indicate diminished leucine and isoleucine catabolism, potentially reflecting a difference in dietary intake in KTRs (32), or changes in mitochondrial oxidative processes. MMA is also produced in part by gut microbial propionate metabolism (33–35), which raises the additional possibility that post-transplant changes in gut microbial activity may contribute to its reduction. It is important to note, however, that the present cross-sectional design does not allow mechanistic inference.

Several dicarboxylic acids including adipic, glutaric, and 3-hydroxysebacic acids were conversely elevated in KTRs. High urinary levels of these dicarboxylic acids have previously been associated with oxidative stress in membranous nephropathy (36), and glutaric acid has been identified as a urinary marker for allograft injury (37). Several other microbiome-derived metabolites were differentially abundant between groups, including hippuric acid, catechol, 4-hydroxybenzoic acid and other aromatic and phenolic metabolites. While shifts in gut microbial composition after transplantation have been documented (38, 39), these differences could equally reflect dietary changes, immunosuppression, or chronic medication use. Any microbiome-related interpretation remains hypothetical in the absence of microbiome data.

Among all enriched pathways, glyoxylate and dicarboxylate metabolism carried the highest topological impact, driven primarily by glycolic acid. Hallan et al. reported similar patterns in the same pathway in CKD patients (21), while Dong et al. identified it as the strongest discriminator of graft injury (40). Further supporting its clinical relevance, Post Hospers et al. (41) found that higher pre-transplant plasma glycolic acid levels were associated with a lower risk of delayed graft function, itself a determinant of long-term graft survival. The consistency across cohorts and clinical contexts suggests a potentially meaningful signal that may carry prognostic information in the transplanted kidney.

Overall, the metabolites identified in this study are significantly and consistently altered in stable KTRs compared to healthy donors and may inform non-invasive monitoring strategies. The enrichment of pathways related to the TCA cycle and glyoxylate metabolism identifies metabolic domains that could be targeted for future therapeutic or nutritional interventions. Moreover, the baseline metabolic profile established here provides a reference against which injury-specific or outcome-specific metabolic changes can be compared and interpreted in future targeted metabolomics studies. However, as this is an exploratory cross-sectional study, causality cannot be established and requires future mechanistic experiments.

Several steps would need to be undertaken to advance these findings toward clinical and biological relevance. First, longitudinal studies with pre- and post-transplant sampling within the same individuals would be relevant to understand the temporal relationship between transplantation and the observed metabolic changes. Second, comparison with matched non-transplant CKD controls would help disentangle metabolic changes attributable to reduced kidney function from those specific to the transplant process. Third, investigating the top discriminant metabolites for associations with specific graft injuries or clinical outcomes would clarify their clinical relevance. Fourth, extension to multi-center cohorts would provide insights on the reproducibility and generalizability of these findings. Finally, extending the analytical platform to other biological compartments, including serum or plasma, could provide a more global and comprehensive view of the post-transplant metabolic phenotype.

### Limitations

Several limitations merit consideration. The cross-sectional design precludes causal inference and residual confounding despite covariate adjustment cannot be ruled out. Additionally, the urinary metabolome of stable KTRs likely represents an aggregate picture, which is, in part, shaped in part by chronic immunosuppressive therapy. Although this means that individual metabolite changes cannot be attributed solely to the transplant process itself, it does not diminish the value of this benchmark. Mapping the metabolic profile of stable transplant recipients provides a reference that can aid in the interpretation of future stratified studies, especially when metabolic differences between disease states may have modest effect sizes despite having clinically meaningful ramifications. Additionally, chronic immunosuppression is, by definition, part of the clinically realized post-transplant state that this study aims to characterize. Still, overall, the biological interpretations discussed above should be viewed as hypothesis-generating. Moreover, residual non-transplant-specific factors, such as dietary patterns, variation in muscle mass and post-transplant care may also influence urinary metabolite levels independently of transplant-specific biology and should be taken into consideration when interpreting the metabolic signature of KTRs.

## Conclusion

In this cross-sectional study, we identify covariate-independent metabolic alterations in the urine of stable kidney transplant recipients compared to healthy living kidney donors, among which the TCA cycle, glyoxylate and dicarboxylate metabolism, and BCAA catabolism are the most prominently perturbed pathways. These findings are consistent with alterations in oxidative metabolism as a feature of the post-transplant metabolic phenotype. Future studies are warranted to elucidate causal vs. correlational frameworks and establish contributions from reduced renal function, chronic medication use, and comorbidity.

## Data Availability

The data concern health care data of kidney transplant recipients. Even in a pseudonymized dataset, individual patients would be recognizable based on the combination of parameters age, sex, eGFR, medication use, transplantation date, donor age and donor sex. The pseudonymized data can be shared upon reasonable request from individual researchers, provided a data transfer agreement is in place between our institution and the institution of the requesting researcher.

## TransplantLines Investigators

Coby Annema, Stephan J.L. Bakker, Stefan P. Berger, Hans Blokzijl, Frank A.J.A. Bodewes, Marieke T. de Boer, Kevin Damman, Martin H. de Borst, Isabelle J.C. Dielwart, Arjan Diepstra, Gerard Dijkstra, Caecilia S.E. Doorenbos, Rianne M. Douwes, Michele F. Eisenga, Michiel E. Erasmus, C. Tji Gan, Antonio W. Gomes Neto, Eelko Hak, Bouke G. Hepkema, Jip Jonker, Frank Klont, Tim J. Knobbe, Daan Kremer, Henri G.D. Leuvenink, Willem S. Lexmond, Vincent E. de Meijer, Hubert G.M. Niesters, Gertrude J. Nieuwenhuijs-Moeke, L. Joost van Pelt, Robert A. Pol, Anna M. Posthumus, Adelita V. Ranchor, Jan Stephan F. Sanders, Marion J. Siebelink, Riemer H.J.A. Slart, J. Casper Swarte, Daan J. Touw, Marius C. van den Heuvel, Coretta van Leer-Buter, Marco van Londen, Charlotte A. te Velde Keyzer, Erik A.M. Verschuuren, Michel J. Vos, Rinse K. Weersma.
