# Supplementary material for: Benchmarking the urinary transplant metabolome of kidney transplant recipients using healthy organ donors
Source: Front Transplant. 2026 Jun 3;5:1833811. doi: 10.3389/frtra.2026.1833811 (PMC13272301; doi:10.3389/frtra.2026.1833811)
Supplement: Supplementary file 1 [file Table1.docx]

Supplementary Materials

# Supplementary Tables

**Table S1**

| **Table S1**. Quality control (QC) coefficients of variation (CV) Two pooled reference urine samples (QC Low and QC High) were analyzed across the full measurement period with n = 21 injections per QC level. Concentrations are expressed in µmol/mmol creatinine. | | | | |
| --- | --- | --- | --- | --- |
| **Metabolite** | **QC Low mean** | **QC Low CV (%)** | **QC High mean** | **QC High CV (%)** |
| Glycolic acid | 15.64 | 12.6 | 31.23 | 14.8 |
| 3-Hydroxypropionic acid | 3.70 | 11.6 | 38.76 | 12.1 |
| p-Cresol | 53.34 | 16.3 | 106.87 | 15.8 |
| 3-Hydroxybutyric acid | 0.63 | 18.5 | 58.62 | 6.1 |
| 3-Hydroxyisobutyric acid | 4.40 | 10.4 | 26.08 | 10.3 |
| 3-Hydroxyisovaleric acid | 2.09 | 16.0 | 8.83 | 15.6 |
| 4-Hydroxybutyric acid | 0.82 | 12.3 | 4.35 | 11.6 |
| Methylmalonic acid | 1.26 | 9.0 | 14.86 | 6.8 |
| Ethylmalonic acid | 2.06 | 20.8 | 6.68 | 14.6 |
| Glyceric acid | 1.94 | 15.9 | 14.63 | 14.5 |
| Succinic acid | 10.75 | 12.3 | 30.26 | 6.7 |
| Methylsuccinic acid | 2.17 | 9.3 | 3.91 | 7.9 |
| Fumaric acid | 0.61 | 7.0 | 10.88 | 4.3 |
| Glutaric acid | 0.54 | 13.4 | 59.69 | 12.8 |
| 3-Methylglutaric acid | 0.49 | 13.2 | 2.60 | 12.2 |
| 3-Methylglutaconic acid (mean) | 2.55 | 10.7 | 6.74 | 9.4 |
| Malic acid | 0.95 | 5.2 | 19.20 | 9.2 |
| Adipic acid | 1.60 | 11.2 | 10.20 | 10.5 |
| 3-Hydroxyglutaric acid | 0.54 | 25.1 | 2.74 | 21.0 |
| 2-Hydroxyglutaric acid | 2.06 | 17.5 | 9.52 | 14.5 |
| 3-Hydroxy-3-methylglutaric acid | 1.96 | 19.3 | 16.27 | 15.2 |
| Isovalerylglycine | 0.79 | 9.8 | 18.03 | 7.5 |
| 2-Oxoglutaric acid | 12.59 | 19.4 | 31.37 | 19.3 |
| Succinylacetone (mean) | 0.30 | 10.3 | 2.36 | 7.8 |
| Tiglylglycine | 1.80 | 9.0 | 3.85 | 9.1 |
| Suberic acid | 1.07 | 15.8 | 4.62 | 14.0 |
| Hexanoylglycine | 0.30 | 0.0 | 4.24 | 4.3 |
| cis-Aconitic acid | 21.51 | 12.6 | 47.96 | 15.2 |
| N-Acetylaspartic acid | 7.22 | 6.8 | 25.01 | 8.5 |
| Citric acid | 279.27 | 18.3 | 320.72 | 22.7 |
| Methylcitric acid (mean) | 3.81 | 19.1 | 74.96 | 14.5 |
| Sebacic acid | 0.11 | 26.8 | 6.71 | 14.2 |
| Vanillactic acid | 0.10 | 0.0 | 0.74 | 14.7 |

# Supplementary Figures

**Figure S1**

**
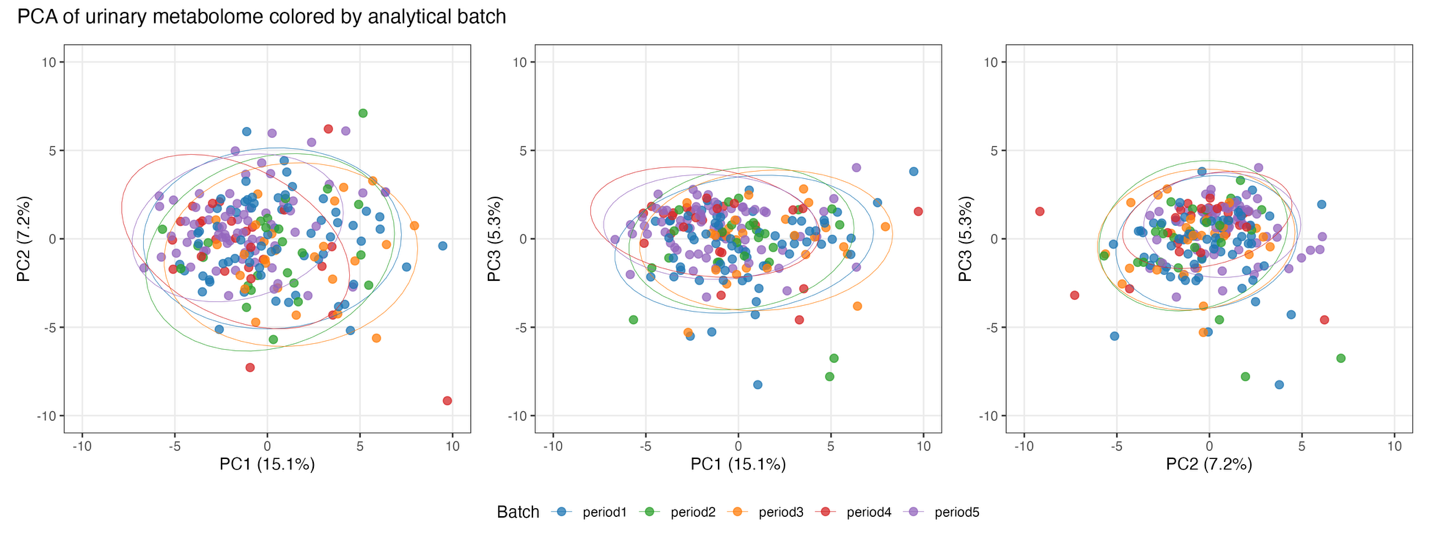
**

**Figure S1**. Principal component analysis of urinary metabolite profiles of all 215 participants, colored by analytical batch. Panels show PC1 vs. PC2, PC1 vs. PC3, and PC2 vs. PC3. Ellipses represent 95% confidence regions per batch. No meaningful batch-related clustering is observed.

**Figure S2**


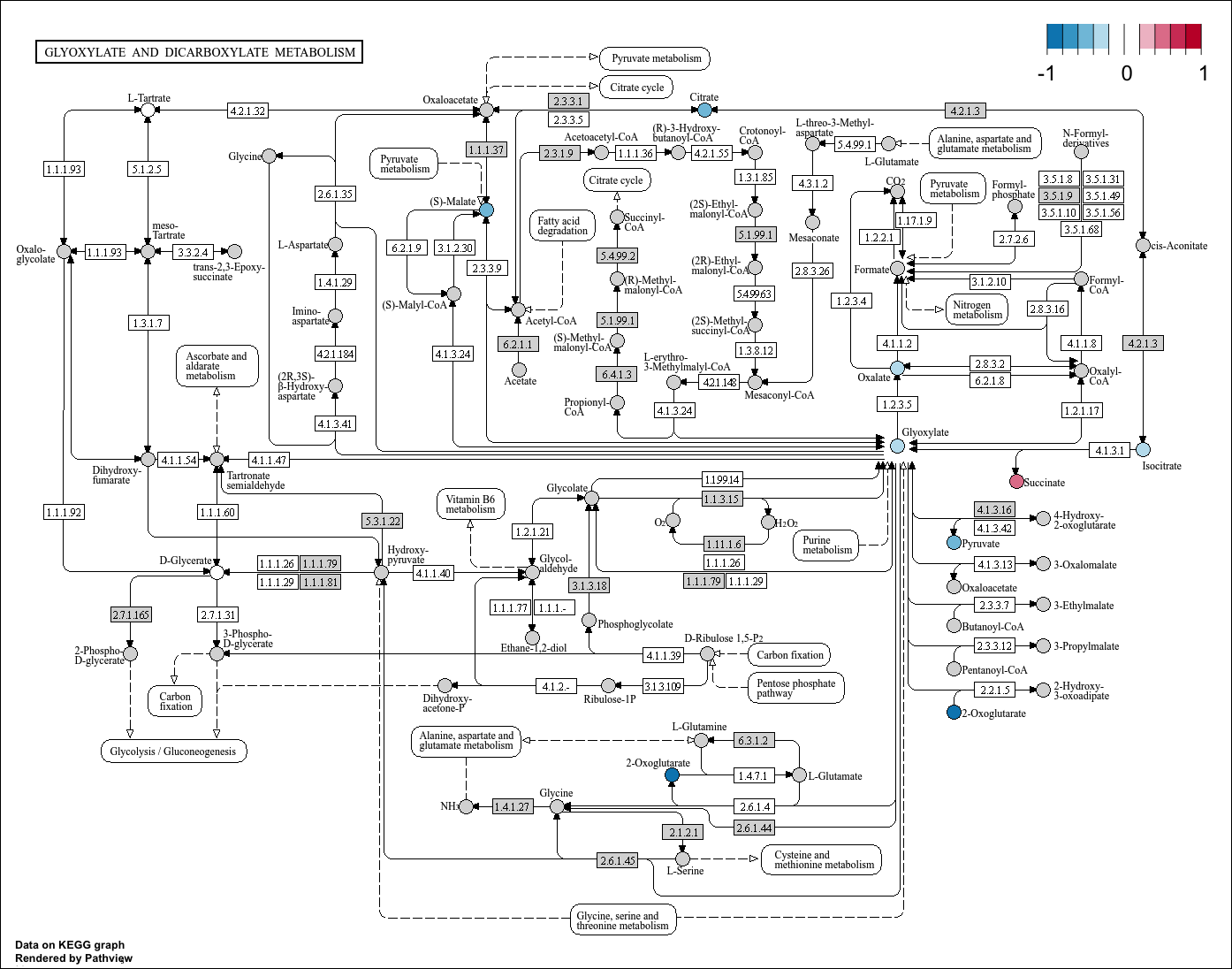


**Figure S2**. KEGG pathway map for glyoxylate and dicarboxylate metabolism. Metabolites are highlighted red when increased in KTRs, and blue when increased in HC.

**Figure S3**

**
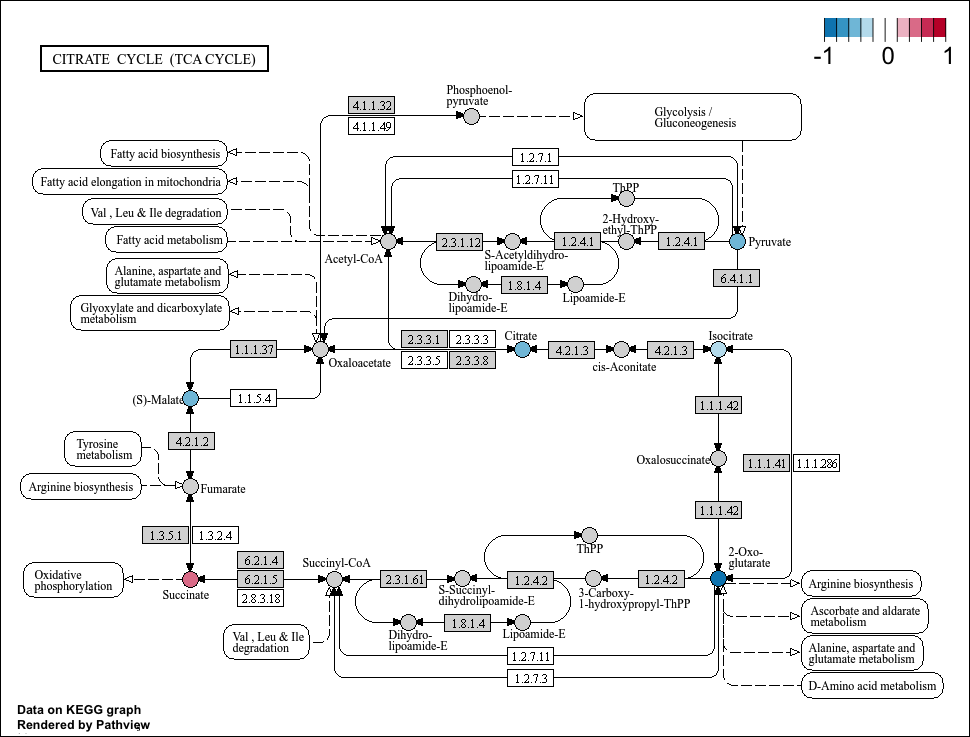
**

**Figure S3**. KEGG pathway map for the TCA cycle. Metabolites are highlighted red when increased in KTRs, and blue when increased in HC.

**Figure S4**

**
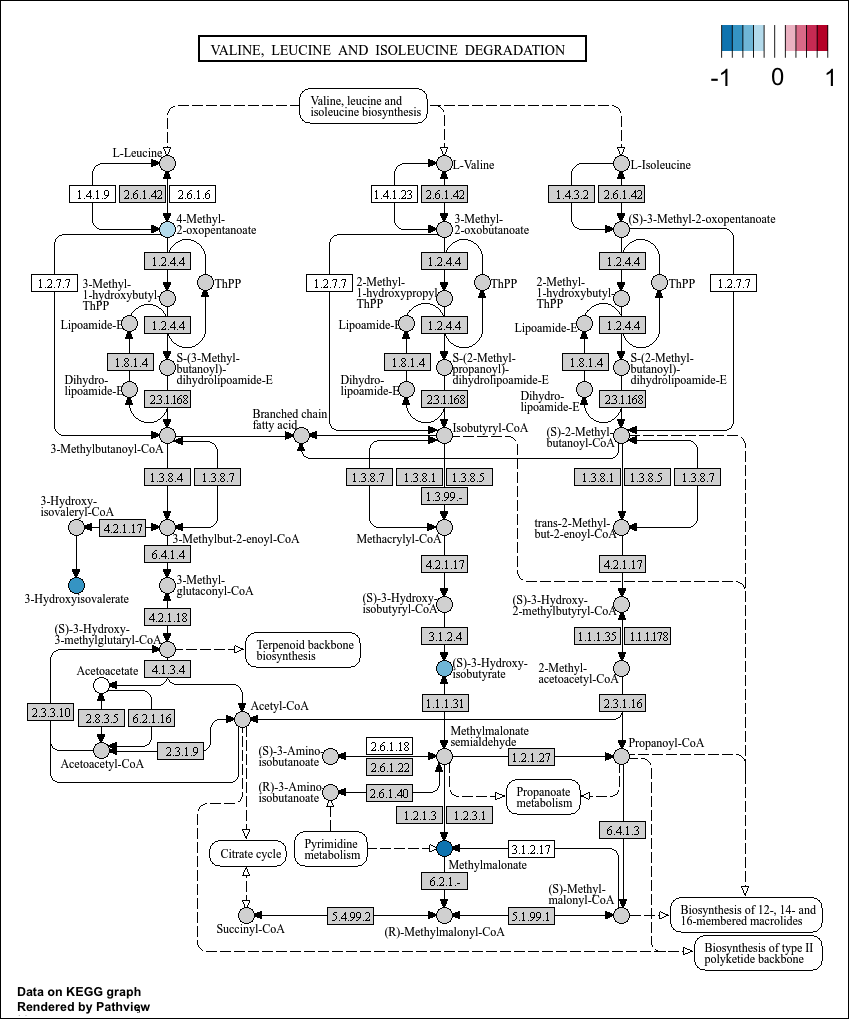
**

**Figure S4**. KEGG pathway map for valine, leucine and isoleucine (BCAA) degradation. Metabolites are highlighted red when increased in KTRs, and blue when increased in HC.
